# History-dependent muscle resistance to stretch remains high after small, posturally relevant pre-movements

**DOI:** 10.1242/jeb.245456

**Published:** 2023-09-27

**Authors:** Brian C. Horslen, Gregory N. Milburn, Kyle P. Blum, Surabhi N. Simha, Kenneth S. Campbell, Lena H. Ting

**Affiliations:** ^1^ Department of Kinesiology and Health Sciences, University of Waterloo, Waterloo, ON, Canada, N2L 3G1; ^2^ Wallace H. Coulter Department of Biomedical Engineering, Emory University and The Georgia Institute of Technology, Atlanta, GA 30332, USA; ^3^ Department of Physiology, University of Kentucky, Lexington, KY 40536, USA; ^4^ Department of Physiology, Feinberg School of Medicine, Northwestern University, Chicago, IL 60611, USA; ^5^ Department of Rehabilitation Medicine, Division of Physical Therapy, Emory University, Atlanta, GA 30322, USA

**Keywords:** Muscle cross-bridges, Muscle thixotropy, Short-range stiffness, Postural sway, Single muscle fiber

## Abstract

The contributions of intrinsic muscle fiber resistance during mechanical perturbations to standing and other postural behaviors are unclear. Muscle short-range stiffness is known to vary depending on the current level and history of the muscle's activation, as well as the muscle's recent movement history; this property has been referred to as history dependence or muscle thixotropy. However, we currently lack sufficient data about the degree to which muscle stiffness is modulated across posturally relevant characteristics of muscle stretch and activation. We characterized the history dependence of muscle's resistance to stretch in single, permeabilized, activated, muscle fibers in posturally relevant stretch conditions and activation levels. We used a classic paired muscle stretch paradigm, varying the amplitude of a ‘conditioning’ triangular stretch–shorten cycle followed by a ‘test’ ramp-and-hold imposed after a variable inter-stretch interval. We tested low (<15%), intermediate (15–50%) and high (>50%) muscle fiber activation levels, evaluating short-range stiffness and total impulse in the test stretch. Muscle fiber resistance to stretch remained high at conditioning amplitudes of <1% optimal fiber length, *L*_0_, and inter-stretch intervals of >1 s, characteristic of healthy standing postural sway. An ∼70% attenuation of muscle resistance to stretch was reached at conditioning amplitudes of >3% *L*_0_ and inter-stretch intervals of <0.1 s, characteristic of larger, faster postural sway in balance-impaired individuals. The thixotropic changes cannot be predicted solely on muscle force at the time of stretch. Consistent with the disruption of muscle cross-bridges, muscle resistance to stretch during behavior can be substantially attenuated if the prior motion is large enough and/or frequent enough.

## Teaser

**Summary:** Intrinsic muscle fiber resistance to stretch is preserved after small, slow pre-movements based on healthy postural sway, but markedly reduced as pre-movements increase to emulate abnormal postural sway.

## INTRODUCTION

Mechanical properties of contractile muscle, in concert with neural control mechanisms, are important mediators of postural control of the limbs and body (Nichols and Houk, 1976, 1973). When a muscle is stretched during standing balance as a result of mechanical perturbations to the body, it can take tens to thousands of milliseconds for the nervous system to initiate and complete a postural correction (Carpenter et al., 2005; Horak, 2006; Ivanenko and Gurfinkel, 2018; Ting et al., 2009). As a result of these relatively long sensorimotor delays, the initial stabilization of the body in response to a perturbation depends on resistive forces from intrinsic muscle mechanical properties (De Groote et al., 2017; Ivanenko and Gurfinkel, 2018; Nichols and Houk, 1973; Ting et al., 2009). Muscle stiffness – a metric for estimating muscle's intrinsic resistance to stretch – varies depending on the current level and history of muscle activation, as well as the muscle's recent movement history (Campbell and Moss, 2002). However, we currently lack sufficient data about the degree to which muscle stiffness is modulated across posturally relevant characteristics of muscle stretch and activation. Therefore, the goal of this study was to characterize muscle's intrinsic resistance to stretch in single muscle fiber experiments at muscle stretch and activation levels relevant to human standing postural sway in health and disease.

**Fig. 1.**
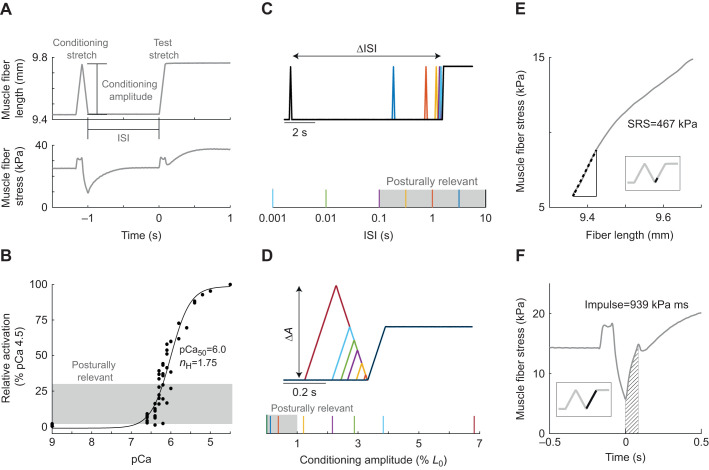
**Experimental design: muscle fiber stretch patterns, activation and outcome measures.** (A) Exemplar data illustrating the muscle stretch response during the paired conditioning–test stretch protocol for a fiber activated to 12.5% maximum in pCa 6.4 solution (where pCa=−log_10_[Ca^2+^]). Muscle fiber length was changed to impose a ‘conditioning’ stretch–shorten cycle of variable amplitude followed by a ramp and hold ‘test’ stretch, with a variable inter-stretch interval (ISI). Modulation of muscle resistance was characterized in the test stretch. (B) Muscle fiber activation level versus Ca^2+^ concentration of activating solution for all fibers included in the dataset (*n*=11). Relative activation was calculated as a percentage of isometric force produced in maximally activating pCa 4.5 solution. These data were used to identify the [Ca^2+^] that yielded posturally relevant activation levels (2–30% maximum), shaded in gray. Single-trial samples from all fibers (filled circles) were used to identify Hill equation (Eqn 1) parameters pCa_50_=6.00 and *n*_H_=1.75, representing mean activation characteristics across all fibers in our sample. (C) Variation in ISI, i.e. the duration of the isometric hold between the conditioning stretch–shorten cycle and ramp-and-hold test stretch. Posturally relevant ISI is shaded in gray at the bottom. (D) Variation in conditioning stretch–shorten cycle amplitude (Δ*A*) with constant stretch velocity. Posturally relevant amplitudes are shaded in gray at the bottom. (E) Short-range stiffness (SRS) was calculated as the slope of the muscle fiber stress–strain curve over the first 20 ms of stretch (black dashed line; see inset for corresponding region of the length–time trace). (F) Impulse was calculated as the area under the stress–time curve (hatched region) for the duration of the ramp period (see inset for corresponding region of the length–time trace).

The history dependence of muscle stiffness due to muscle stretch, i.e. muscle thixotropy, has been well characterized in single muscle fiber experiments. When a muscle fiber is stretched, it exhibits a high but transient initial stiffness (stress increase per unit change in muscle fiber length) referred to as short-range stiffness (SRS). If the muscle continues to be stretched beyond this ‘short range’, then the stiffness decreases (Rack and Westbury, 1974). History-dependent variations in SRS have been demonstrated in single muscle fibers using a paired stretch paradigm where a conditioning stretch–shorten cycle immediately precedes a test stretch (Fig. 1A) (Herbst, 1976; Lakie and Campbell, 2019; Lakie and Robson, 1988; Lännergren, 1971). A conditioning stretch–shorten cycle of sufficient amplitude (often ∼3% optimal fiber length, *L*_0_) reduces SRS in a second, test stretch of the same amplitude by up to ∼50% (Campbell and Moss, 2002; Lännergren, 1971). If the inter-stretch interval (ISI) between the conditioning and test stretch is increased, the SRS in the test stretch increases monotonically up to at least 10 s before fully recovering its stiffness (Campbell and Moss, 2002; Rassier and Herzog, 2004).

Much of the knowledge on muscle thixotropy comes from experiments conducted at relatively high levels of muscle activation that do not focus on lower, posturally relevant muscle activation levels. During quiet standing, plantar flexor muscles are typically activated between 5% and 20% of maximum voluntary activation in healthy and impaired adults, respectively (Warnica et al., 2014). While larger history-dependent reductions in muscle SRS have been demonstrated at intermediate activation levels (∼20–50% maximum activation) (Campbell and Moss, 2002), most muscle fiber thixotropy studies focus on 50–100% activation levels so as to maximize signal-to-noise ratio, or suit other experimental needs (Altman et al., 2015; Lynch et al., 2008). Therefore, in the current study, we focused on the effects of activation on muscle thixotropy when activated below 50%.

Further, prior experiments have not investigated muscle thixotropy at small, posturally relevant conditioning stretch amplitudes. In healthy standing balance, the ankle dorsiflexors and plantar flexors are critical for maintaining upright posture and are continuously stretched and shortened as the body sways (Day et al., 2013; Loram et al., 2009). Postural sway-induced ankle muscle stretch during unperturbed standing is estimated to be less than 1% *L*_0_ in healthy adults (Day et al., 2013; Ivanenko and Gurfinkel, 2018; Loram et al., 2009). However, postural sway is faster and larger in individuals with impaired balance (Horak and Macpherson, 2011; Maurer et al., 2003); increases in postural sway of up to 2 times that of healthy individuals (Maurer et al., 2003) suggest that muscle stretch could reach up to 2% in individuals with balance impairments (Loram et al., 2009). Thus, understanding the effects of conditioning amplitudes between 1% and 3% *L*_0_ may be critical to understanding how muscle mechanical contributions to balance control may differ across healthy and impaired conditions.

Based on the biophysical mechanisms underlying muscle force generation, we predicted that muscle resistance to stretch would be modulated by posturally relevant timing and amplitudes of the conditioning stretch at low activations typical of standing balance control (Lakie and Campbell, 2019). Briefly, muscle force can be considered to arise from both the number and length of bound actin–myosin muscle cross-bridges. Muscle stiffness arises from the resistance of the attached cross-bridges, such that the greater the number of attached cross-bridges, the greater the muscle stiffness. Consequently, muscle SRS arises when the number of bound cross-bridges is high, such as when the muscle is held isometrically. However, muscle stiffness decreases rapidly when sarcomeres are stretched beyond a few nanometers, causing myosin heads to be pulled off their actin binding sites, rapidly reducing the number of bound cross-bridges. Therefore, pre-stretching a muscle can decrease the number and length of bound cross-bridges, reducing muscle stiffness and force in subsequent stretches. Conversely, increasing the ISI during which the muscle is held isometric allows cross-bridges to reattach over time, increasing muscle resistance to stretch in subsequent stretches. Therefore, the muscle resistance to stretch depends on the net effect of prior stretch and rest intervals.

While studies on the mechanisms of history dependence in muscle resistance to stretch focus on SRS, the muscle's force throughout a stretch provides additional information about its role in movement. SRS has been used to characterize muscle thixotropy but describes only the muscle's resistance at the onset of muscle stretch. Behaviorally, the success of a muscle in rejecting a postural perturbation depends on its resistive force throughout stretch, regardless of the muscle stiffness. Impulse, measured as the time integral of force, is proportional to the change in momentum imparted to an inertial load, and may provide better insight into the role of history-dependent muscle resistance to stretch on its role in postural behaviors.

**Fig. 2.**
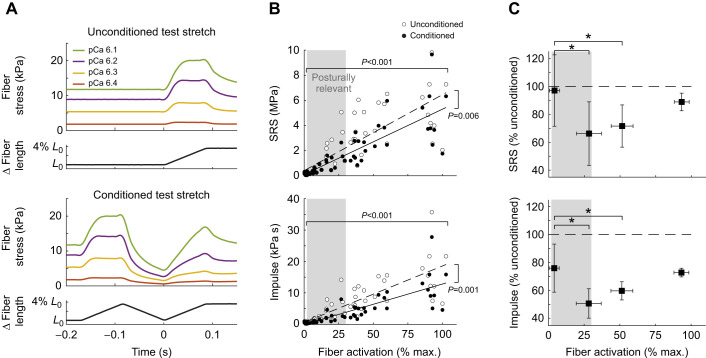
**Effect of activation level on thixotropic changes to muscle fiber resistance to stretch.** (A) Representative data from a single permeabilized muscle fiber demonstrate that muscle fiber stress increased with activation level (compare colors) in both unconditioned (top) and conditioned trials (bottom) with a 3.83% *L*_0_ (where *L*_0_ is optimal fiber length) conditioning stretch and 0.001 s ISI. (B) Across all fibers (*n*=11), SRS (top) and impulse (bottom) increased monotonically with increasing muscle fiber activation (slope of line: *P*<0.001) for both unconditioned and conditioned stretches. However, SRS of unconditioned stretches increased faster than that of conditioned stretches (slope of line: *P*=0.005). (C) Relative decreases in both SRS (top) and impulse (bottom) between conditioned (black squares) and unconditioned trials (dashed horizontal line at 100%) were largest at intermediate activation levels. Means±s.d.; horizontal bars with asterisks represent significant differences (*P*<0.05) between activation levels; gray boxes represent posturally relevant activation ranges (2–30% maximum).

**Fig. 3.**
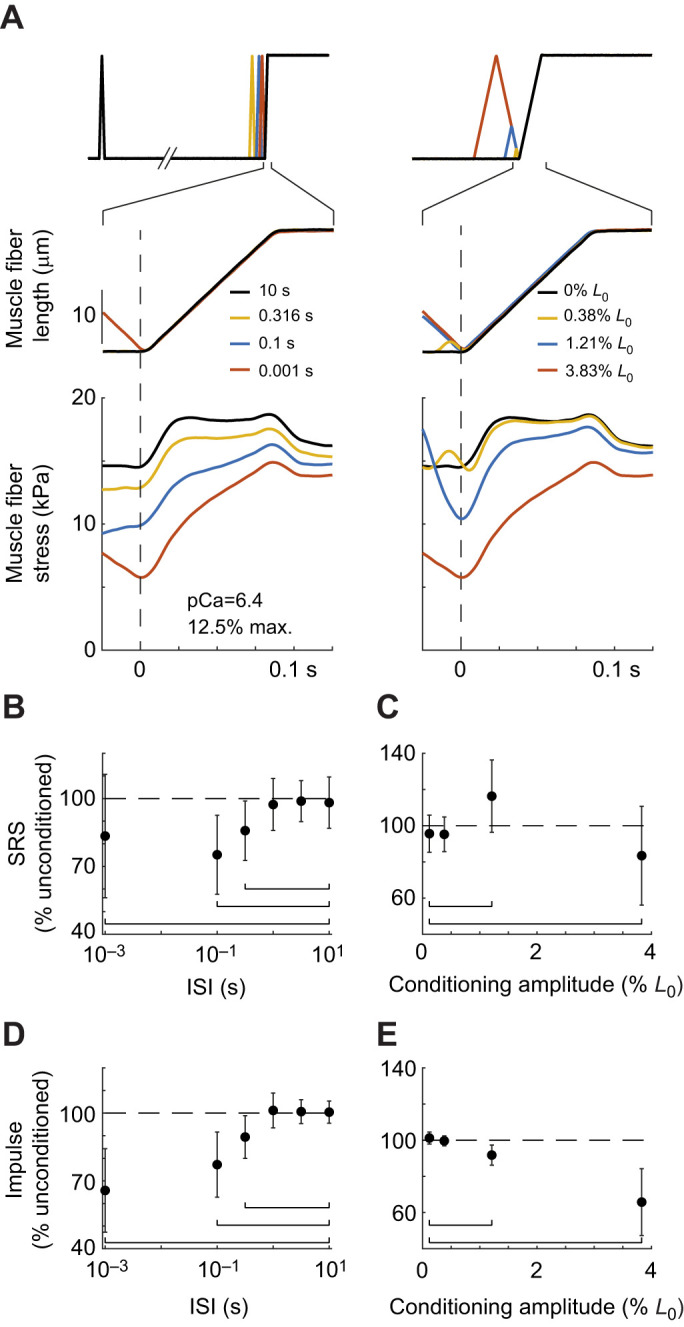
**Independent evaluation of ISI and conditioning stretch amplitude effects on muscle fiber stiffness.** (A) Representative data of muscle fiber stress at low activation (12.5%, pCa 6.4) showing the independent effects of decreasing ISI when conditioning amplitude is constant (3.83% *L*_0_; left), and increasing conditioning stretch amplitude when ISI is constant (0.001 s; right). Traces are aligned in time to onset of the test stretch (dashed vertical lines), which was identical in velocity and displacement across all trials. The SRS in conditioned test stretches was greatest when ISI was longest (10 s; left, black) and decreased with shorter ISIs (compare colors). Conversely, SRS was greatest when the conditioning amplitude was smallest (0% *L*_0_; right, black) and decreased as conditioning amplitude increased (compare colors). Across all fibers, the percentage unconditioned SRS was lowest at (B) short ISIs and (C) large conditioning stretch amplitudes. Horizontal brackets indicate statistically significant (*P*<0.05) contrasts between sample and 10 s ISI value. Likewise, the percentage unconditioned impulse was lowest when (D) ISI was short or (E) conditioning amplitude was large. Horizontal brackets indicate statistically significant (*P*<0.05) contrasts between sample and 0.12% *L*_0_ conditioning amplitude value. Means±s.d., *n*=11.

Here, we characterized the history dependence of single muscle fiber resistance to stretch in posturally relevant ranges of ISI, muscle activation and conditioning stretch amplitude. We tested rat soleus muscle fibers which – similar to human soleus muscles – are almost all Type I slow-twitch muscles in adulthood (Larson et al., 2019). Rat soleus muscle fibers exhibit greater history dependence and are more robust to long experimental protocols compared with faster muscle fibers (Campbell and Moss, 2002; Ramsey et al., 2010). We activated permeabilized muscle fibers and used a paired conditioning–test stretch design where a balance perturbation-like ‘test’ stretch was preceded by a postural sway-like stretch–shorten ‘conditioning’ cycle (Fig. 1A). We systematically varied: (1) bath Ca^2+^ concentration to alter muscle activation level (Fig. 1B), (2) the ISI between conditioning stretch–shorten cycles and the test stretch (Fig. 1C) and (3) the amplitude of conditioning stretches (Fig. 1D). We used the same, constant velocity for all stretches. We characterized muscle resistance to stretch in two ways: we estimated (1) SRS at the onset of stretch (Fig. 1E) and (2) overall resistance of the muscle to stretch, calculated as total impulse throughout the duration of the test stretch (Fig. 1F). Overall, we found that muscle resistance to stretch is least affected by prior movement at lower postural muscle activation and in stretch conditions more characteristic to healthy postural sway, and reduced most by prior movement at intermediate levels of postural activation and larger conditioning stretch amplitudes more similar to sway in balance impairments.

## MATERIALS AND METHODS

### Ethical approval

Eleven soleus muscle fibers harvested from 2 adult female Sprague–Dawley rats (Envigo, Indianapolis, IN, USA; RRID: RGD_737903) were studied. Experiments were performed at The University of Kentucky. Animals were housed in clean cages with a 12 h light/dark cycle in an animal care facility and had access to food and water *ad libitum*. Experimental procedures were reviewed and approved by the University of Kentucky IACUC (no. 00784M2004; assurance no. D16-00217 [A3336-01]).

### Sample harvest and preparation

Procedures and solutions used for muscle fiber harvest, permeabilization and storage, as well as equipment and procedures used to conduct experiments are described in detail elsewhere (Campbell, 2006; Campbell and Moss, 2002). In brief, rats were anesthetized by intraperitoneal injection of pentobarbital (50 mg kg^−1^ body mass) and subsequently killed by surgical excision of the heart, and the soleus muscles were harvested immediately, teased into fiber bundles, then permeabilized for 4 h. Permeabilized muscle fiber bundles were stored in a glycerol solution at −20°C for no more than 1 month prior to use.

### Experimental apparatus

Single muscle fibers were tied between a motor (model 312, Aurora Scientific Inc., Aurora, ON, Canada) and force transducer (model 403, Aurora Scientific Inc.). Muscle fiber length was controlled, and length and force data recorded, using SLControl software (www.slcontrol.com; Campbell, 2006); both data sampling and length position update rates were set to 1 kHz. Initially, muscle fiber length was manually adjusted under a microscope and in minimally activating solution (see below) to a mean sarcomere length of 2.6 µm (assumed optimal fiber length, *L*_0_). This fiber length was recorded, and all subsequent length manipulations were scaled as a fraction of this *L*_0_.

### Characterizing muscle fiber activation

After establishing *L*_0_, we incrementally increased each muscle fiber bath Ca^2+^ concentration to characterize the fiber's full range of activation levels (Fig. 1B). Permeabilized fibers were chemically activated using free Ca^2+^ solutions with concentrations ranging from pCa (=−log_10_[Ca^2+^]) 9.0 (minimal Ca^2+^) to pCa 4.5 (maximal), with densest sampling in the pCa 6.6–6.0 range (Fig. 1B). pCa solutions contained 20 mmol l^−1^ imidazole, 14.5 mmol l^−1^ creatine phosphate, 7 mmol l^−1^ EGTA, 4 mmol l^−1^ MgATP, 1 mmol l^−1^ free Mg^2+^, free Ca^2+^ ranging from 1 nmol l^−1^ to 32 μmol l^−1^ and KCl to adjust ionic strength to 180 mmol l^−1^ with pH 7.0 at 22°C; a 22°C mean temperature was maintained for all experiments. At each activation level we calculated mean isometric stress over 0.9 s with the fiber held at a mean sarcomere length of 2.6 µm (*L*_0_). We then calculated the stress as a percentage of pCa 4.5 isometric stress and took this value as the achieved activation level. We fitted data for all fibers to a sigmoidal curve to estimate the mean Hill equation parameters, *n*_H_ and pCa_50_ (pCa required to achieve 50% activation), for our sample (Fig. 1B) (Eqn 1) (Donaldson and Kerrick, 1975). We estimated that pCa_50_=6.00 and *n*_H_=1.75:
(1)

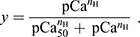


### Identifying posturally relevant activation levels

After characterizing muscle fiber Ca^2+^ sensitivity, we selected at least three pCa solutions to test the effects of muscle fiber activation level on history-dependent muscle resistance to stretch. pCa solutions were selected to yield low, intermediate and high activation levels relative to muscle activation in standing balance tasks (2–30%) (Warnica et al., 2014). Low activation was defined as less than 15% maximum (where maximum is isometric force at pCa 4.5); intermediate was 15–40% maximum; and high activation was greater than 40% maximum. To preserve the viability of the muscle fibers, activation levels were tested in ascending order, where all conditioning amplitude and ISI combinations were tested (cf. ‘Posturally relevant ISIs’ and ‘Posturally relevant conditioning amplitudes’, below) before moving to the next activation level. Activation levels were verified *post hoc* and used to assign groups for statistical comparison. Not all fibers yielded data in each activation range and some fibers yielded more than one dataset within a range. If fiber appearance or mechanical properties had not deteriorated after stretching at high activation (i.e. striations remained clear under the microscope and isometric force was not changed by more than 20% from initial activation) (Campbell and Moss, 2002), then the fiber was maximally activated in pCa 4.5 solution. At pCa 4.5, the muscle was stretched for as many trials as possible before degradation (usually 2–3 stretches) to create a maximal activation dataset.

### Paired conditioning and test stretch protocol

To characterize the modulation of muscle resistance to stretch, ISI and conditioning stretch–shorten amplitude were systematically varied (Fig. 1C,D, cf. ‘Posturally relevant ISIs’ and ‘Posturally relevant conditioning amplitudes’, below), while analyses were performed on the test stretch (Fig. 1E,F). Ramp and hold characteristics of the test stretch were fixed across all trials (amplitude: 3.83% *L*_0_, velocity: 45.45% *L*_0_ s^−1^, hold duration: 2 s); these parameters were selected for behavioral relevance to postural perturbations. We tested 25 unique ISI and conditioning amplitude combinations in 11 fibers. This included a test stretch with no preceding conditioning stretch to establish a baseline response at each activation level. In 3 of the 11 fibers, we also included an additional 25 combinations to further explore interactions between ISI and conditioning amplitude (cf. ‘Posturally relevant ISIs’ and ‘Posturally relevant conditioning amplitudes’).

### Posturally relevant ISIs

ISI between the conditioning and test stretches was varied along a logarithmic scale between 0.001 s and 10 s (Fig. 1A,C). This range of ISIs (0.001, 0.1, 0.316, 1, 3.162, 10 s) was selected considering known ISI-dependent changes in muscle stiffness (Campbell and Moss, 2002, 2000; Proske and Stuart, 1985) and the range of human postural sway frequencies (0.1–5 Hz; Fig. 1C, gray shading) (Peterka, 2002; Prieto et al., 1996). In a sub-set of fibers (3/11), we included a sample at a 0.01 s ISI to increase the resolution of time-dependent effects.

### Posturally relevant conditioning amplitudes

We systematically varied the conditioning stretch amplitude in the paired stretch protocol (Fig. 1A,D). Conditioning stretch velocity was fixed at 45.45% *L*_0_ s^−1^, matching the test stretch velocity. Based on pilot studies, we anticipated a logarithmic scaling of responses with conditioning stretch amplitude, causing us to sample a range from 0.12% *L*_0_ to 3.83% *L*_0_ (Fig. 1D; 0.12%, 0.38%, 1.21%, 3.83% *L*_0_). This range encompasses very small stretches that are less than the short-range elastic limit (0.2% *L*_0_) (Hill, 1968), small amplitudes observed in healthy postural sway (<1% *L*_0_; Fig. 1D, gray shading), amplitudes used in previous muscle fiber mechanics studies (3% *L*_0_) (Campbell and Moss, 2002), and large, dynamic amplitudes that occur in reaching or gait (>3% *L*_0_). In a sub-set of fibers (*n*=3/11), we added trials with conditioning amplitudes of 2.16%, 2.88% and 6.82% *L*_0_ to improve resolution and to better establish the interacting effects of conditioning stretch interval and amplitude.

### Muscle resistance outcome measures

We quantified history-dependent changes in muscle fiber resistance to stretch by calculating SRS and impulse of the test stretch in each trial. SRS refers to the initial stiffness of the muscle in response to stretch and was calculated as the slope of the stress versus strain curve in the first 0.02 s after the onset of test stretch (Fig. 1E). Impulse refers to the time-integral of force and characterizes the total resistance of the muscle throughout the test stretch, well beyond the initial period where SRS is computed. Impulse was computed as the area under the stress versus time curve for the duration of the lengthening phase of the ramp stretch (0.084 s) (Fig. 1F).

Additionally, we isolated the response to the test stretch by removing the conditioning transient to determine whether history-dependent changes in muscle fiber stiffness were distinct from, or a product of, recovery in muscle fiber stress following conditioning. We therefore isolated the effect of the test stretch by subtracting the force transient of the conditioning stretch alone from the total stress (see Fig. 6A for an example in a representative muscle fiber). The recorded stress (Fig. 6A top, thick black trace) drops below pre-conditioning levels following the first, conditioning stretch and does not return to the original stress level at the onset of the test stretch. We measured the stress transient for each conditioning stretch amplitude, at each activation level, and for each muscle fiber in the 10 s ISI condition (Fig. 6A, gray trace). The isolated test stress was computed by subtracting the conditioning transient from the recorded stress for each ISI for the matching conditioning amplitude and activation level for each fiber (Fig. 6A, red trace). We then re-calculated SRS and impulse from the computed test stress.

### Statistical analyses

All datasets and R software used in this study can be found at https://osf.io/3jy62/?view_only=4f09424a1c5d468798baa1cf8d673100 (supplementary materials and methods). We performed an initial assessment of the effects of muscle fiber activation level on history-dependent changes in SRS and impulse. SRS and impulse were computed for unconditioned test stretches across activation levels, as well as test stretches conditioned with a 3.83% *L*_0_, 0.001 s ISI at all activation levels. Such large-amplitude, short-interval conditioning patterns have previously been shown to yield large reductions in SRS across a range of fiber activation levels (Campbell and Moss, 2002). Separate linear mixed models were used to test whether SRS or impulse varied with activation and conditioning stretch. In each model, activation was treated as a continuous variable and conditioning was a dichotomous variable (conditioned versus unconditioned), and each fiber (*j*) was included as a random variable:
(2)

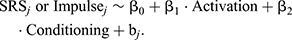
We first tested the effects of activation on absolute levels of SRS and impulse and compared these effects in conditioned versus unconditioned test stretches. We further tested the effect of activation on relative changes in SRS (% unconditioned test stretch) and impulse. Here, we used categorical levels of fiber activation of low (<15% maximum), intermediate (15–40% maximum), high (40–60% maximum) and maximum (>60% maximum) as independent variables, and the relative change in conditioned versus unconditioned SRS and impulse (% unconditioned) as dependent variables in the linear mixed model. In all our analyses, we report the coefficient values, *t*-values (estimate/standard error) and *P*-values. Analyses were performed in R software using ‘lmerTest::lmer’ routines.

**Fig. 4.**
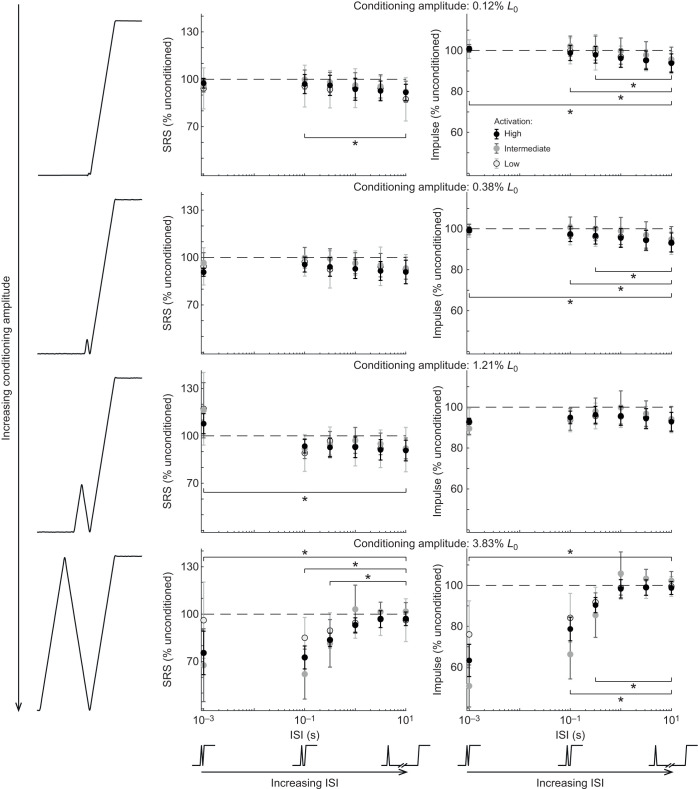
**Interactions between conditioning stretch amplitude and ISI across activation level.** Each panel shows the effects of increased ISI at three activation levels for a single conditioning stretch amplitude on mean SRS (left) and impulse (right). From top to bottom, each row represents progressively larger conditioning stretch amplitudes. At small conditioning stretch amplitudes (top three rows), there was little absolute change in either SRS or impulse with increasing ISI. As conditioning stretch amplitude increased, both SRS and impulse were reduced at the smallest ISIs (bottom row). Horizontal dashed lines indicate 100% unconditioned stretch SRS or impulse values. Horizontal brackets and asterisks indicate statistically significant differences from the 10 s ISI condition, collapsed across activation conditions. Means±s.d., *n*=11.

We next tested how ISI, conditioning amplitude and muscle fiber activation level affect history-dependent changes in relative SRS or impulse (Fig. 4). We computed SRS and impulse for all test stretches, and expressed them as a percentage of the unconditioned test stretch, to obtain relative SRS and impulse values. On the full dataset (*n*=11 fibers), we used linear mixed models in R software (lmerTest::lmer) with ISI (6 levels: 0.001, 0.1, 0.316, 1, 3.162, 10 s), conditioning amplitude (4 levels: 0.12% *L*_0_, 0.38% *L*_0_, 1.21% *L*_0_, 3.83% *L*_0_) and activation level (3 levels: low, intermediate, high) coded as categorical variables, fiber as a random variable, and the dependent measures, SRS or impulse, as continuous variables:
(3)

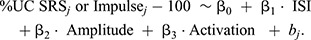
The maximum activation level did not yield enough ISI and conditioning amplitude combinations to be included as a distinct group in this analysis; as such, these data were included in the ‘high’ group. The reference level was set to be the trials with the smallest conditioning amplitude (0.12% *L*_0_), longest ISI (10 s) and lowest activation level (<15% maximum).

We used *post hoc* contrasts to test our specific hypotheses about the effects of ISI and conditioning amplitude on history-dependent changes in SRS and impulse (Fig. 3A, bottom row). Estimated marginal means contrast tests (R software, emmeans::emmeans) were used to isolate main effects from the linear mixed model results as outlined in the above paragraph.

To isolate the effect of ISI, we limited the analysis to the large, 3.83% *L*_0_, amplitude because small conditioning stretches often failed to change SRS or impulse (Fig. 4) (Lännergren, 1971). We collapsed the data across all activation levels and fixed conditioning amplitude at 3.83% *L*_0_, then compared change in SRS or impulse (percentage unconditioned) marginal means across ISIs. To isolate the effect of conditioning amplitude on SRS or impulse, we again collapsed the data across activation levels and fixed ISI at 0.001 s, then compared SRS or impulse (percentage unconditioned) marginal means across amplitudes. Similar to ISI above, we limited the analysis to the short, 0.001 s, ISI because long ISIs have been shown to fail to change SRS (Campbell and Moss, 2002). The threshold for statistical significance was set to 0.05; *P*-values were adjusted using Tukey's method for multiple comparisons (ISI: 6 estimates; conditioning amplitude: 4 estimates).

We performed similar analysis on the set of samples with higher sample density (*n*=3) to further characterize ISI and conditioning amplitude interaction effects. As above, we used separate linear mixed models (lmerTest::lmer) to test for effects of activation and conditioning parameters on SRS and impulse. In these tests, we included categorical variables ISI (7 levels: 0.001, 0.01, 0.1, 0.316, 1, 3.162, 10 s), conditioning amplitude (7 levels: 0.12% *L*_0_, 0.38% *L*_0_, 1.21% *L*_0_, 2.16% *L*_0_, 2.88% *L*_0_, 3.83% *L*_0_, 6.82% *L*_0_) and activation level (3 levels: low, intermediate, high), and muscle fiber as a random variable. Again, the reference level was set to be the trials with the smallest conditioning amplitude (0.12% *L*_0_), longest ISI (10 s) and lowest activation level (<15% maximum).

We further tested whether thixotropic changes in SRS and impulse can be explained solely by the reductions in muscle fiber stress caused by the conditioning stretch. Linear mixed models were used to test whether conditioning stretch amplitude and/or ISI are necessary to explain the variance in our stiffness measures in addition to the instantaneous stress at onset of test stretch. We used linear mixed models (R software; lmerTest::lmer) to test whether SRS or impulse scale as a function of ISI (6-level categorical variable: 0.001, 0.1, 0.316, 1, 3.162, 10 s), conditioning amplitude (4-level categorical variable: 0.12% *L*_0_, 0.38% *L*_0_, 1.21% *L*_0_, 3.83% *L*_0_), instantaneous stress at onset of test stress (in kPa as continuous variable) and muscle fiber as a random variable:
(4)

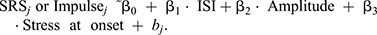


## RESULTS

### Effect of activation on the history dependence of muscle fiber resistance to stretch

Consistent with previous studies (Campbell and Moss, 2002), muscle resistance to stretch increased with muscle activation level in both unconditioned and conditioned test stretches and was lower in conditioned trials. Muscle stress changed in amplitude over time during the test stretch as bath calcium concentration increased (see example in Fig. 2A, colored traces) in both unconditioned and conditioned test stretches. However, there was a qualitative change in the shape of the muscle stress in response to the test stretch in conditioned (Fig. 2A, top) compared with unconditioned (Fig. 2A, bottom) responses. These qualitative changes in muscle stress trajectories reflect a history-dependent change in muscle resistance to stretch. Across all fibers in our sample, both SRS and impulse increased with activation (Fig. 2B; SRS: β=57.7, *t*_107.3_=17.9, *P*<0.001; impulse: β=0.159, *t*_107.3_=17.6, *P*<0.001). However, both SRS and impulse were significantly greater in unconditioned stretches than in conditioned ones when considered across all activations (SRS: β=557.3, *t*_105_=2.8, *P*=0.006; impulse: β=2.21, *t*_105_=3.9, *P*<0.001).

The relative reduction in muscle resistance to stretch in the conditioned trials was non-linear as a function of activation level, with the greatest reductions at posturally relevant intermediate activation levels (Fig. 2C, gray shaded region). Although there were significant differences in the slope of the linear analyses, the relative changes in muscle resistance to stretch appear to be greater at intermediate levels of activation (Fig. 2B, open versus filled black circles). When activation was binned into low (<15% maximum), intermediate (15–40% maximum), high (>40–60% maximum) and maximal activation levels (>60% maximum), the conditioned SRS and impulse expressed as a percentage of the unconditioned level exhibited a U-shaped relationship to activation (Fig. 2C). SRS was not decreased from unconditioned levels in the low activation bin (β=3.54, *t*_23.9_=−0.76, *P*=0.456), but was further decreased (Fig. 2C, top) in the intermediate (β=−29.2, *t*_46.8_=−4.4, *P*<0.001) and high (β=−23.3, *t*_49.3_=−2.8, *P*=0.008) activation bins, compared with low activation; maximal activation SRS was not different from low activation values, when expressed as a percentage of unconditioned values. Impulse was decreased in the low activation condition, compared with unconditioned levels (β=24.1, *t*_30.5_=−9.19; *P*<0.001), and further decreased (Fig. 2C, bottom) in both the intermediate (β=−25.8, *t*_47.59_=−6.1, *P*<0.001) and high activation (β=−15.8, *t*_48.1_=−3.0, *P*=0.004) bins. Impulse, as a percentage of unconditioned stretch values, was not different from low activation levels in the maximal activation bin.

### Independent evaluation of the effects of ISI and conditioning amplitude on muscle resistance to stretch

We observed similar qualitative changes in muscle stress trajectories during the test stretch due to both variations in ISI and conditioning amplitude (see example in Fig. 3A of a fiber at 12.5% activation). Consistent with prior reports, muscle stress during the test stretch was greatest at the longest ISI of 10 s when large conditioning amplitudes (3.83% *L*_0_) were used (Fig. 3A, left, black traces), resembling muscle stress when there was no conditioning stretch (0% *L*_0_; Fig. 3A, right, black trace). Muscle stress during the test stretch decreased systematically when either ISI of the large conditioning stretch was decreased (Fig. 3A, left, colored traces) or conditioning stretch amplitude at 1 ms ISI was increased (Fig. 3A, right, colored traces). In both cases, stress decreased both at the onset of the test stretch (dashed vertical line) and for the duration of the lengthening period. Note that the conditions where the stress was lowest represent the same trial, where the ISI was 0.001 s and conditioning stretch amplitude was 3.83% *L*_0_ (Fig. 3A red traces in left and right panels).

Across all fibers and activation levels, significant reductions in muscle resistance to stretch were found when decreasing ISI while holding conditioning stretch amplitude constant. The reduction of both the percentage unconditioned SRS and impulse was lower in the shortest three ISIs (0.001, 0.01 and 0.316 s) compared with the longest (10 s) ISI (Fig. 3B,D). The largest reductions in SRS and impulse occurred at the shortest ISI, where they were both around 70% unconditioned level, and increased with longer ISIs until reaching about 100% unconditioned level at 0.316 s (Fig. 3B,D).

Across all fibers and activation levels, significant reductions in muscle resistance to stretch were also found when increasing conditioning stretch amplitude while holding ISI constant at 1 ms. Large conditioning stretch amplitudes (3.83% *L*_0_) led to significant reductions in SRS (84% unconditioned) and impulse (66% unconditioned), compared with those at the smallest conditioning amplitude (0.12% *L*_0_; Fig. 3C). However, at the next smallest conditioning stretch amplitude of 1.21% *L*_0_, SRS was slightly higher than unconditioned levels, while impulse was slightly lower than unconditioned levels; data from smaller conditioning stretch amplitudes were about 100% unconditioned level.

### Interactions between ISI and conditioning stretch amplitude

Interactions between the effects of ISI and conditioning stretch amplitude were found at all activation levels (Fig. 4). When all conditioning and activation permutations were examined, there were main effects of both ISI (SRS: *F*_5,1283.8_=9.8, *P*<0.001; impulse: *F*_5,1283.8_=42.8, *P*<0.001) and conditioning stretch amplitude (SRS: *F*_3,1283.8_=20.0, *P*<0.001; impulse *F*_3,1283.8_=109.0, *P*<0.001), consistent with the main effects reported above. There was an interaction between ISI and conditioning amplitude on SRS (*F*_15,1283.8_=21.8, *P*<0.001) and impulse (*F*_15,1283.8_=72.0, *P*<0.001). These interactions were visualized by plotting all changes in SRS and impulse as a function of ISI at each of four conditioning stretch amplitudes (Fig. 4). The largest reductions in SRS and impulse were observed when the conditioning stretch amplitude was largest (Fig. 4, bottom). There were also small, but statistically significant, increases in both SRS and impulse at small conditioning amplitudes. While we found an effect of activation level on SRS and impulse with the largest amplitude, shortest interval stretches (Fig. 2C), there was no effect of activation on SRS or impulse when all ISI and conditioning amplitude permutations were considered together (Fig. 4).

### Increased sampling density characterizes transitions from high to low muscle fiber resistance to stretch

**Fig. 5.**
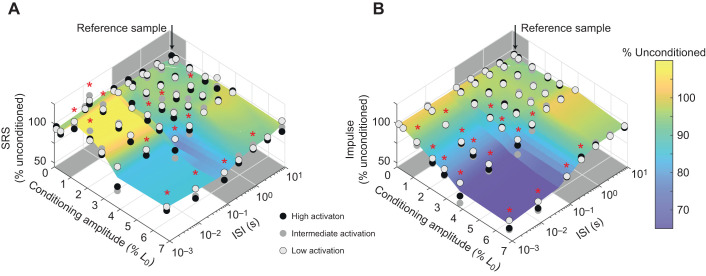
**Increased sampling density reveals transitions in SRS and impulse with increasing conditioning stretch amplitude and decreasing ISI.** The effects of conditioning amplitude (left horizontal axis), ISI (right horizontal axis) and activation level (circles: gray to black) on (A) SRS and (B) impulse as a percentage of the unconditioned response (vertical axis). Gray boxes in both plots indicate posturally relevant ranges of conditioning stretch amplitude (<1% *L*_0_) and ISI (0.1 s to 1 s). Both SRS and impulse decreased monotonically as conditioning amplitude increased between 1% and 3% *L*_0_ but did not decrease further at 6% *L*_0_. This is similar to the non-linear changes in stiffness as ISI decreased, with no additional effects of decreasing the ISI below 0.01 s. *n*=3 fibers; asterisks indicate statistically significant differences from a conditioning amplitude of 0.12% *L*_0_ and ISI of 10 s.

In the dataset above, we lacked data points between amplitudes eliciting significant effects to characterize the transitions in the effect of conditioning stretch amplitude, motivating the acquisition of additional data with finer sampling of conditioning stretch amplitude (Fig. 5).

**Fig. 6.**
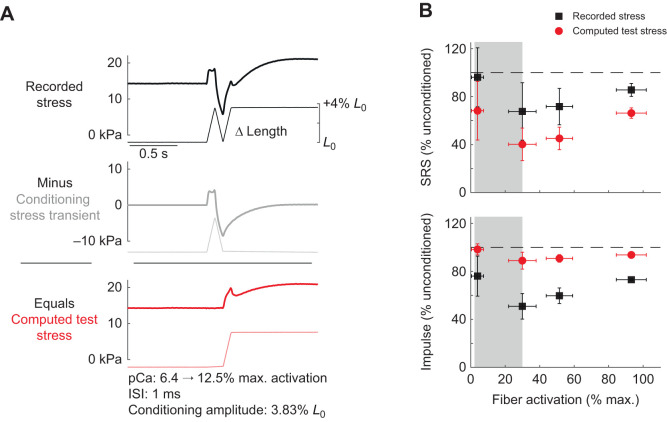
**Removal of conditioning stretch stress transient has quantitative but not qualitative effects on thixotropic changes to SRS and impulse.** (A) Representative single fiber data stress during a conditioned stretch. While the conditioning and test stretches are readily distinguished in the muscle fiber length record (thin black line), the recorded stress (thick black line) includes the effect of the first, conditioning, stretch as well as the second, test, stretch. The force transient (dark gray line) associated with each conditioning stretch (light gray line) was subtracted from the total stress (thick black line) to isolate the stress response (red line) to the test stretch (light red line). (B) Changes in SRS (top) and impulse (bottom), calculated as a percentage of unconditioned stretch values, plotted across activation levels from the 0.001 s ISI, 3.83% *L*_0_ condition. Black squares represent data calculated from recorded stress (replication of Fig. 2C) and red circles represent the same measures calculated from the computed test stress. Thixotropic changes in SRS (top) are relatively larger in the computed test stress than recorded stress (red circles further from 100% unconditioned dashed line than black squares), while changes in impulse are smaller in the computed test stretch than recorded stress, but still reduced from unconditioned levels (red circles below dashed line, but above black squares). Both SRS and impulse from the computed stress follow the same trends as those computed from recorded stress. Means±s.d., *n*=11.

Overall, the more densely sampled conditions in a subset of fibers (3 of 11) revealed a transition between high and low levels of relative resistance to stretch due to interactions between ISI and conditioning stretch consistent with normal and abnormal postural sway and activation (Fig. 5). Specifically, the addition of more conditioning amplitudes between 1% and 7% *L*_0_ and a 0.01 s ISI revealed a monotonic transition between high and low percentage unconditioned SRS (Fig. 5A) and impulse (Fig. 5B), paralleling the reductions seen with varying ISI (Fig. 5, compare left and right horizontal axes).

Accordingly, the percentage unconditioned SRS was dependent upon interactions between ISI and conditioning amplitude (*F*_36,731_=11.6, *P*<0.001), as well as main effects of ISI (*F*_6,731_=36.7, *P*<0.001) and conditioning amplitude (*F*_6,731_=44.1, *P*<0.001). Similar main and interacting effects of ISI and conditioning amplitude were found for impulse (interaction: *F*_36,731_=20.9, *P*<0.001; ISI: *F*_6,731_=111, *P*<0.001; conditioning amplitude: *F*_6,731_=115.8, *P*<0.001).

Within the healthy posturally relevant range where ISIs were greater than 0.1 s and conditioning amplitudes were lower than 1% *L*_0_, both conditioned SRS (Fig. 5A) and impulse (Fig. 5B) remained high, near 100% unconditioned levels (Fig. 5, gray shaded regions). None of these conditions were significantly less than the reference condition of 0.001 s and 0.12% *L*_0_ conditioning stretch. SRS was statistically higher than in the reference condition at the 0.01 s interval, 0.38% *L*_0_ amplitude permutation, which is within the posturally relevant range.

The transition from high to low percentage unconditioned SRS and impulse occurred at combinations of shorter ISIs and higher conditioning amplitudes characteristic of abnormal postural sway. Further, there appeared to be a plateau in the reduction of muscle SRS at combinations of conditioning amplitude and ISI greater than 3% and lower than 0.01 s. The monotonic decrease and plateau effects could be readily observed in SRS across the 0.1 s ISI. SRS was significantly reduced below 0.12% *L*_0_ levels at all conditioning amplitudes greater than or equal to 2.88% *L*_0_ (all *P*<0.05) when ISI was fixed at 0.1 s. SRS was also lower in the 6.82% *L*_0_ condition than at either 2.16% or 2.88% *L*_0_, demonstrating a persistent decrease in stiffness with increasing conditioning amplitude (Fig. 5A). However, there was no difference between SRS levels between the 3.83% and 6.82% *L*_0_ amplitudes, suggesting the effect reached a plateau (Fig. 5A, bottom). Impulse followed a similar pattern of monotonic decreases with increasing conditioning amplitude or decreasing ISI, although no plateau effect was observed for impulse (Fig. 5B).

Finally, on average, SRS as a percentage of unconditioned values was higher in this sub-set of fibers at intermediate levels of activation when all conditioning amplitude and interval permutations were considered (*F*_2,732.4_=45.0, *P*<0.001).

### Thixotropic changes to SRS and impulse persist after removing conditioning stretch force transients

The ramp-and-release conditioning stretch caused transient reductions in muscle fiber stress (Fig. 3A, Fig. 6A black trace) that gradually recovered to pre-conditioning levels over time (Fig. 6A, gray trace). As such, there were length-independent increases in muscle fiber stress following conditioning that, at short ISIs, coincided with the test stretch. We tested whether our measures of muscle stiffness (length-dependent changes in stress) during the test stretch were distorted by the length-independent stress recovery that co-occurred with the test stretch.

The U-shaped relationship between muscle fiber activation and both SRS and impulse persisted when transient conditioning stretch after-effects were removed (Fig. 6B). Fig. 6B shows the SRS (top) and impulse (bottom) from the total recorded stress (black squares) and the computed stress to isolate the effects of the test stretch alone (red circles) in the 0.001 s ISI and 3.83% *L*_0_ amplitude trials across activation levels; black squares in Fig. 6B are the same data as presented in Fig. 2C. Thixotropic changes in SRS of the isolated test stress were greater than those of the total stress across all activation levels (red circles lower than black squares). The U-shaped pattern across activation levels was preserved in SRS from the isolated test stress (distance from dashed horizontal line), with the largest effects at intermediate (15–40% maximum) and high activation levels (40–60% maximum). In contrast, the thixotropic effects on the impulse of the isolated test stress were smaller than those of the total stress (red circles above black squares) but nevertheless exhibited a similar pattern of reduced impulse with conditioning (red circles and error bars both below dashed line), with the largest differences in the conditioned versus unconditioned responses occurring at intermediate and high activation levels.

### Muscle fiber stress at onset of test stretch may underpredict muscle resistance to stretch in history-dependent conditions

**Fig. 7.**
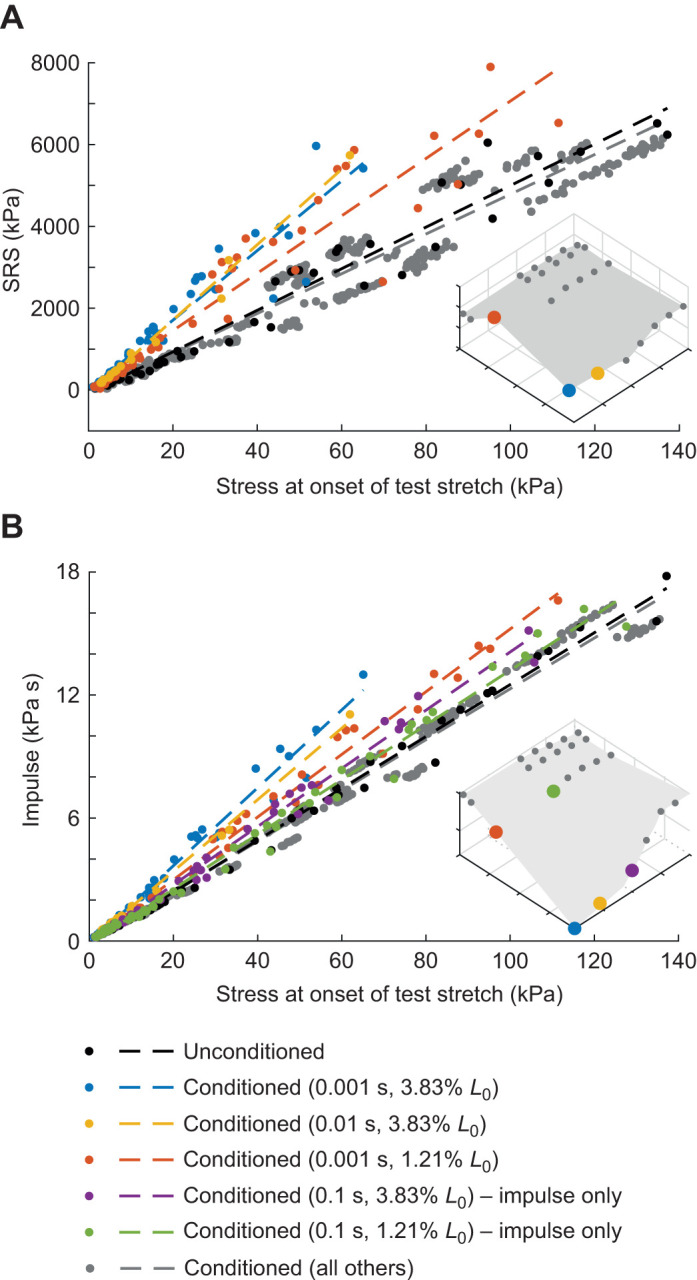
**Stretch history affects the relationship between stress at onset of test stretch and muscle resistance to stretch.** (A) SRS versus muscle fiber stress sampled immediately preceding test stretch. Black circles show SRS calculated from unconditioned data with the dashed black line representing a linear fit. Colored circles and fits correspond to combinations of ISI and conditioning amplitude where significant history-dependent effects were found; gray circles and fits represent all other conditioned data. The slopes of the colored fits are all greater than the unconditioned (black) or conditioned (gray) fits. (B) Impulse versus muscle fiber stress at onset of the test stretch, with the same coloring convention as in A. At short ISIs and large conditioning stretch amplitudes, the slopes of the impulse–stress relationships were greater than at unconditioned (black) or other conditioning parameter combinations (gray). Insets: 3D surface plots aligning colors to surface plots from Fig. 5 for reference.

Muscle fiber stress at the onset of the test stretch was a good predictor of muscle resistance to stretch in both unconditioned stretches (Fig. 7, black) and conditions where history dependence was not present (Fig. 7, gray). Overall, stress at onset of the test stretch was the strongest predictor of SRS (β=48.5, *t*_1262.5_=37.1, *P*<0.001) and impulse (β=0.13, *t*_1263_=95.2, *P* <0.001). However, the slope of the relationship between muscle stress at test stretch onset with both SRS and impulse increased when conditioning stretch amplitude was large and ISI was short. There was a significant effect of an ISI of 0.001 s and a conditioning amplitude of 3.83% *L*_0_ on both SRS (Fig. 7A, blue; β=35.8, *t*_1262_=9.8, *P*<0.001) and impulse (Fig. 7B, blue; β=0.06, *t*_1262_=17.6, *P*<0.001). Reducing conditioning amplitude to 1.21% *L*_0_ at the same 0.001 s ISI halved the increase in slope (Fig. 7, red; SRS: β=21.7, *t*_1262_=8.0, *P*<0.001; impulse: β=0.03, *t*_1262_=10.1, *P*<0.001). A similar halving effect was observed in the impulse with each order of magnitude increase in ISI or decrease in conditioning stretch amplitude. Though there was little effect of increasing stretch interval from 0.001 to 0.01 s at 3.83% *L*_0_ (Fig. 7, yellow), the slope was halved when ISI was increased to 0.1 s at 3.83% *L*_0_ (Fig. 7B, purple; β=0.015, *t*_1262_=5.4, *P*<0.001) and when amplitude was reduced to 1.21% *L*_0_ at 0.1 s (Fig. 7B, green; β=0.006, *t*_1262_=2.4, *P*=0.019). Overall, statistically significant interactions between stress at onset, ISI and conditioning stretch amplitude explained approximately 5–40% of the overall impulse or SRS response to stretch.

## DISCUSSION

Here, we have shown that history-dependent reductions in muscle resistance to stretch in single muscle fibers are absent in posturally relevant activation and movement conditions but present in conditions consistent with abnormally elevated postural sway. We used a classical paired muscle stretch paradigm where a ‘conditioning’ triangular stretch–shorten cycle is followed by a ‘test’ ramp-and-hold. We systematically varied the triangular conditioning stretch amplitudes and ISIs based on muscle stretch amplitudes and frequencies observed in normal and abnormal postural sway. The effects on SRS and impulse during the ramp-and-hold test stretch provided insight about how postural sway may modulate instantaneous muscle resistance to stretch in a balance perturbation (De Groote et al., 2017).

Our data revealed history-dependent changes in muscle resistance to stretch when conditioning stretches occurred at larger and faster amplitudes than those seen in normal postural sway. Consistent with fluctuations in muscle length during standing postural sway in healthy individuals (Warnica et al., 2014), muscle SRS and impulse remained near 100% when the conditioning stretches were below ∼1% fiber length and/or occurred more than ∼1 s prior to the test stretch. These findings were consistent across all muscle activation levels. However, history-dependent reductions in muscle SRS and impulse were progressively observed as the conditioning stretch amplitude increased beyond 1.2% and/or ISIs decreased below 0.1 s. Notably, those with balance impairments are estimated to have ∼2% fluctuations in muscle stretch during postural sway and higher sway frequency (Horak and Macpherson, 2011; Maurer et al., 2003), suggesting reduced muscle resistance to stretch in balance-impaired individuals. A maximum reduction of about 70% nominal SRS and impulse values occurred when conditioning stretches were larger than 3% and occurred earlier than 0.1 s; it is not clear whether this reduction would be reached during continuous movements such as fast running in humans, in which large stretches would occur cyclically at a period of ∼0.3 s per step (Lichtwark et al., 2007). Notably, individuals with poor balance often increase their baseline muscle activity (Martino et al., 2023). Although our data show that intermediate levels of activation (<40% maximum) increased absolute muscle resistance to stretch, there were also greater history-dependent reductions in muscle resistance to stretch.

To our knowledge, this is the first study to characterize the history dependence of muscle resistance to stretch across multiple conditioning amplitudes, and their interaction with ISIs. While prior studies demonstrated a minimum conditioning amplitude for inducing history dependence in muscle fibers (Lakie and Robson, 1988), we revealed a transition region where muscle history dependence was modulated by interactions between conditioning amplitude and ISI. Within the set of experimental conditions tested, a variety of conditioning amplitude and ISI combinations could cause changes in muscle resistance to stretch between 70% and 100% of unconditioned levels. In general, smaller stretches applied at long intervals preserved muscle resistance to stretch, whereas combinations of larger stretch with shorter ISIs reduced muscle resistance to stretch. We did not test different stretch velocities, but based on cross-bridge mechanisms (see below), slower conditioning stretches should tend to preserve muscle resistance to stretch in the test stretch, and faster conditioning stretches would tend to reduce muscle resistance to stretch in the test stretch (Rassier et al., 2003). Although we did not use continuous stretch trajectories, these data suggest that complex movement history of stretched muscle fibers, such as that seen in postural behaviors as well as locomotion, will modulate muscle resistance to stretch.

To increase the behavioral relevance of our findings, we used two metrics of muscle resistance to stretch: SRS and impulse. Effects of muscle history dependence to stretch have typically been studied using SRS or peak force (Campbell and Moss, 2002). Both muscle stiffness and peak force characterize a single point in time during the stretch, so neither metric represents the overall force during a stretch. Behaviorally, the success of a muscle in rejecting a postural perturbation depends on its overall effect throughout a perturbation. The time integral of muscle force, i.e. impulse, represents the total force during the stretch and provides two insights relevant for sensorimotor control. Impulse directly reflects the change in momentum that the force causes on the body that it acts upon. Though not true in general, for our specific conditions using the same test stretch amplitude and velocity, differences in the muscle's impulse and total work (calculated as force over distance) across conditions are proportional. Such impulse- and work-based metrics have been critical in assessing muscle function in movement (Daley and Biewener, 2006; Dickinson et al., 2000). To facilitate the behavioral implications of history dependence in muscle resistance to stretch, we first examined the total resistance at the time of the test stretch, including any transient effects of the conditioning stretches. Later analysis removing these transients served primarily to interrogate the underlying mechanisms of the history dependence.

The changes in muscle resistance to stretch based on movement history are consistent with predictions based on disrupting muscle cross-bridges. Briefly, muscle consists of elastic cross-bridges where myosin heads attach to actin sites to produce force. Muscle force is based on the number and length of attached cross-bridges. However, muscle stiffness depends only on the number of attached cross-bridges, each of which acts as a spring when stretched. As it is possible to achieve the same muscle force with different numbers of attached cross-bridges, the SRS during a stretch cannot be fully predicted based on the level of force at the time of stretch (Fig. 7). Movement history needs to be considered because the conditioning stretches shift the distribution of cross-bridge lengths (Campbell and Lakie, 1998). In conditions of history-dependent reductions in muscle resistance to stretch, both stiffness and force level are reduced compared with the unconditioned stretch responses at the same activation level. Sufficiently large stretch amplitudes cause myosin heads to be unbound from actin, reducing muscle resistance to stretch. But, as ISI increases, the cross-bridges begin to reattach and, over time, this restores the muscle's resistance to stretch. As myosin heads must attach to actin to form cross-bridges, activation level can also affect the history dependence of the muscle fiber. The number of available actin sites also affects both muscle force and the rate of recovery to steady-state length. At very low activation, there are not enough attached cross-bridges for the amplitude and ISI conditionings to have much effect (Fig. 2C). At intermediate activation, more cross-bridges are attached, but the re-attachment to actin will depend on the proximity to an available binding site, causing slower force recovery after stretch. In contrast, at very high activation levels, there may be an abundance of actin sites available for cross-bridges to re-attach after stretch, reducing the effect of the conditioning stretch that disrupts the number and length of attached cross-bridges (Fig. 2C). Accordingly, we found a U-shaped relationship between muscle activation level and the history dependence of muscle resistance to stretch. We further showed that the history-dependent changes in muscle resistance to stretch observed cannot be attributed solely to the level of muscle stress at the time of stretch; instead, it also reflects shifts in the cross-bridge distribution due to prior movement history. A more detailed mechanistic explanation of the biophysical mechanisms underlying history-dependent changes in muscle force can be found in a review by Lakie and Campbell (2019).

Although our manipulations of muscle activation and conditioning stretches were based on the literature in human postural control, there are a number of limitations when using isolated, permeabilized rat muscle fibers to infer the role of muscle properties on behavior. Skinned, isolated, permeabilized fibers are typically quite similar in mechanics, enabling characterization with a relatively small number of fibers (Campbell and Moss, 2002, 2000), which we did comprehensively in each fiber across conditioning amplitudes and ISIs. As both rat and human soleus muscles are primarily slow-twitch, we expect quantitative but not qualitative differences in the relationships in the history dependence of muscle resistance to stretch. However, forces acting on a human muscle–tendon unit during standing depend on a range of other soft tissue properties that will differ across species, including muscle and tendon architecture, mechanical contributions from the extracellular matrix and non-contractile proteins, as well as the muscle fiber type. These anatomical differences may affect the conditions in which history dependence will be behaviorally relevant. Likewise, the continuous low and uniform chemical activation used in this study allowed us to manipulate the level of activation in fine detail, but it is unclear how the intermittent and unequal pulsatile activation seen across a whole muscle in behavior would affect muscle resistance to stretch. Furthermore, slow fibers used in standing balance could be active closer to 50% but would form a small percentage of all muscle fibers, making our findings here still relevant for postural control. Single muscle fiber data were also recorded at a relatively low temperature (22°C) to preserve fiber integrity; while there may be quantitative differences at body temperature, we expect the relative effects on conditioned versus unconditioned muscle resistance to stretch to be similar. Finally, holding muscle fibers isometric prior to, and between, stretches is an artificial state that rarely occurs during behavior; we did not explicitly test the effects of continuous muscle stretching and shortening on muscle resistance to stretch.

Despite these limitations, our results may nonetheless offer some mechanistic insight into the role of pre-movement on the modulation of muscle properties in the context of balance control as well as other postural behaviors including those in the upper limb (Hu et al., 2011; Mathew and Crevecoeur, 2021). First, our data suggest that normal postural sway or movement variability is likely small enough that it does not induce history-dependent reductions in muscle resistance to stretch. The instantaneous mechanical resistance that muscle can provide is a critical first line of defense to stabilize the body before the slower, neurally mediated balance feedback corrections, even at low muscle activation levels (Ivanenko and Gurfinkel, 2018; Ting et al., 2009). Accordingly, recent work shows resistive joint torque during balance perturbations occurring prior to sensorimotor feedback mediates changes in muscle activation occurring at ∼100 ms latency (De Groote et al., 2017; Van Wouwe et al., 2021). Presumably in healthy individuals, greater muscle resistance to stretch in the first 100 ms would reduce the need for neural feedback for balance control.

Overall, our data suggest that conditions of increased postural sway and muscle activation seen in balance impairments decrease the relative contributions of mechanical stabilizing mechanisms from muscle, likely increasing the need to rely on neural feedback control mechanisms for balance. Based on our data, increased postural sway consistent with that seen in balance-impaired individuals would reduce muscle resistance to stretch by up to 30%. Less mechanical stabilization of balance would be available for those with abnormally high postural sway, and necessitate greater reliance on sensorimotor feedback for balance. Indeed, recent evidence shows that large, imposed, sway-like movements decrease ankle stiffness when people stand on a tilting platform (Sakanaka et al., 2016). However, increased muscle activity observed in balance-impaired individuals may lead to a smaller decrease in postural sway because of the greater history-dependent effects of muscle resistance to stretch in conditions similar to abnormal postural sway (>1%, <1 s ISI) at intermediate muscle activation levels. People also tend to increase muscle activity to decrease postural sway in threatening contexts such as standing at heights (Carpenter et al., 2001), when balance is evaluated by a clinician (Geh et al., 2011) or in response to arousing stimuli (Horslen and Carpenter, 2011). This increase in ‘postural stiffness’ could be mediated by absolute changes in muscle resistance to stretch in conditions consistent with normal postural sway (<1% *L*_0_, >1 s ISI). As the history-dependent properties of muscle also contribute to the generation of sensory signals (Blum et al., 2020; 2017; Proske et al., 1993) driving balance-correcting muscle activity (Lockhart and Ting, 2007; Welch and Ting, 2009), the neural response to balance perturbation is likely also diminished under conditions of increased postural sway. As such, the history-dependent properties of muscle resistance to stretch at elevated postural sway levels could negatively impact both mechanical and neural contributions to balance control.
